# Targeted Metabolome Profiling of Indonesian Shallots and Japanese Long-Day/Short-Day Bulb Onions

**DOI:** 10.3390/metabo12121260

**Published:** 2022-12-14

**Authors:** Kanako Matsuse, Mostafa Abdelrahman, Nur Aeni Ariyanti, Fumitada Tsuji, Sho Hirata, Tetsuya Nakajima, Muneo Sato, Masami Yokota Hirai, Benya Manochai, Masayoshi Shigyo

**Affiliations:** 1 Division of Yamaguchi University and Kasetsart University Joint Master’s Degree Program in Agricultural and Life Sciences, Yamaguchi University, Yamaguchi 753-8515, Japan; 2 Molecular Biotechnology Program, Faculty of Science, Galala University, Suze 34511, Egypt; 3 Botany Department, Faculty of Science, Aswan University, Aswan 81528, Egypt; 4 Department of Biology Education, Faculty of Mathematics and Natural Sciences, Universitas Negeri, Yogyakarta 55281, Indonesia; 5 Institute of Food Sciences and Technologies, Ajinomoto Co., Inc., 1-1 Suzukichō, Kawasaki 210-8681, Japan; 6 Laboratory of Agroecology, Department of Bioresource Sciences, Faculty of Agriculture, Kyushu University, 744 Motooka, Nishi-ku, Fukuoka 819-0395, Japan; 7 Laboratory of Vegetable Crop Science, Division of Life Science, Graduate School of Sciences and Technology for Innovation, Yamaguchi University, Yamaguchi 753-8515, Japan; 8 RIKEN Center for Sustainable Resource Science, 1-7-22 Suehiro-cho, Yokohama 230-0045, Japan; 9 Department of Applied Biosciences, Graduate School of Bioagricultural Science, Nagoya University, Nagoya 464-8601, Japan; 10 Department of Horticulture, Faculty of Agriculture, Kasetsart University, Bangkok 10900, Thailand

**Keywords:** bulb onions, metabolite profiling, shallots, sulfur assimilation

## Abstract

In this study, targeted metabolome analysis was applied to identify the discriminative metabolites between Indonesian shallot landraces, Japanese long-day onion (LDO) varieties, and Japanese short-day onion (SDO) varieties. In total, 172 metabolite signal intensities were subjected to multivariate PLS-DA, VIP, and random forest modeling to gain further insight into genotype-specific metabolites. PLS-DA divides the examined genotypes into three different clusters, implying that shallot landraces exhibited a distinct metabolite profile compared with Japanese LDO and SDO varieties. The PLS-DA, VIP, and random forest results indicated that the shallot and LDO are richer in metabolite constituents in comparison with the SDO. Specifically, amino acids and organosulfur compounds were the key characteristic metabolites in shallot and LDO genotypes. The analysis of S-alk(en)yl-L-cysteine sulfoxide (ACSO) compounds showed higher accumulation in the shallot landraces relative to LDO and SDO varieties, which explains the stronger pungency and odor in shallots. In addition, the LDO showed higher ACSO content compared with the SDO, implying that long-day cultivation might enhance sulfur assimilation in the Japanese onion. The LDO ‘Super Kitamomiji’ and the shallots ‘Probolinggo’ and ‘Thailand’ showed higher ACSO content than other varieties, making it useful for *Allium* breeding to improve the flavor and stress tolerance of onions.

## 1. Introduction

*Allium* is one of the largest genera in the family Amaryllidaceae, with approximately 800–900 species that compromise several important vegetable crops, such as garlic (*A. sativum),* onions (*A. cepa*), Japanese bunching onions (*A. fistulosum*), leeks (*A. ampeloprasum*), and shallots (*A. cepa* Aggregatum group) [1,2]. *Allium* species are widely grown across the northern hemisphere, mainly in North America, North Africa, Europe, and Asia, a region with diverse ecological stretches that led to the development of an astonishing number of *Allium* species with various physiological and morphological traits [3]. S-alk(en)yl-L-cysteine sulfoxide (ACSO) and flavonoid compounds are the most characteristic ingredients in *Allium* species and are principally responsible for the species-specific flavor and biological activities [4]. 

In Southeast Asian countries, shallots (2*n* = 16), which are genetically closer to the bulb onion (2*n* = 16), have been recognized as a potential genetic resource for *Allium* crop improvement because of their adaptability to a wide range of environmental stresses [5,6]. Shallots have their own distinct texture and flavor that is somewhat different from that of bulb onions. For example, shallots grow as cluster of bulbs with an elongated shape, whereas onions grow as an individual bulb per plant with a more circular shape [7]. Additionally, shallots produce more flavonoid, ACSO, polysaccharide, and amino acid content than bulb onions [8,9,10]. In contrast, bulb onions produce more monosaccharide content than shallots [11]. Although shallots and bulb onions differ in appearance, color, taste, and phytochemical composition, they have similar nutritional and medicinal properties, and several varieties of bulb onions and shallots have been used as food seasonings, raw vegetables, or in folk medicine for a long time [12,13].

Shallots are commonly used as a condiment in Southeast Asian countries, including Indonesia, Malaysia, Vietnam, and the Philippines, whereas the bulb onions are widely used as fresh vegetables or processed food in Europe, America, Africa, and East Asia, including China and Japan. In Japan, there are two types of bulb onion cultivars, short-day and long-day onions (SDOs and LDOs, respectively) [14], according to their photoperiod requirements for bulb formation, as suggested by Brewster [15]. LDOs need about 14 to 15 h of daylight to bulb, whereas SDOs need 10 h of daylight to bulb [15]. A Japanese seed company developed three different F1 varieties, ‘Okhotsk 222′ (early season), ‘Kitamomiji 2000′ (mid-season), and ‘Super Kitamomiji’ (late season), as LDOs suitable for the Hokkaido area. By using these three types through half of the year, farmers can harvest bulb onions from early to late September. In the same way, seven different F_1_ varieties were developed as SDOs for the southwestern part of Japan and can be harvested continuously from late April to early June, before the rainy season [11]. These leading varieties can be cultivated predominantly in the main bulb onion–production areas from north (Hokkaido) to south (Awaji and Saga) in Japan. Even so, no one knows the metabolomic profiles of the two complete sets for Japanese LDO and SDO varieties. Since the metabolic profiles were unknown for these eight Indonesian shallot landraces, three Japanese LDO varieties, and seven Japanese SDO varieties, we assumed that the profiles were the same among each group and the same within each group. Thus, further research using omics technology could be beneficial for exploiting the metabolite characteristics between Indonesian shallot landraces and Japanese SDO and LDO varieties. In this study, targeted metabolome profiling of eight Indonesian shallot landraces, three Japanese LDO varieties, and seven Japanese SDO varieties was investigated using liquid chromatography–tandem quadrupole mass spectrometry (LC-QqQ-MS). Our results provide useful information regarding shallot–bulb onion metabolic traits, which are significant for use in the production of an F_1_ hybrid between shallots and bulb onions to improve the taste and flavor as well as the stress tolerance of bulb onions.

## 2. Materials and Methods

### 2.1. Plant Materials

**Figure 1:**
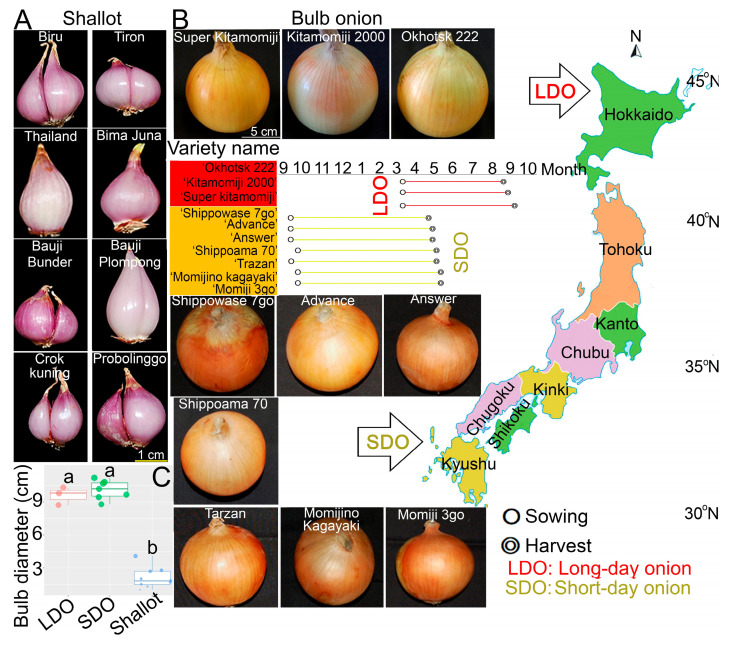
Phenotypes of the investigated Indonesian shallot landraces and Japanese long-day and short-day onions (LDO and SOD, respectively) as well as bulb diameter (cm). (**A**,**B**) phenotypes of eight Indonesian shallot landraces and three Japanese LDO and seven SDO varieties. (**C**) Bulb diameters of the investigated genotypes. Values represent means calculated from three independent replicates (*n* = 3). Different letters indicate significantly higher bulb diameter of LDO and SDO compared with shallots using Tukey’s honestly significant difference (HSD) test.

In this study, three Japanese LDO, seven Japanese SDO varieties, and eight Indonesian shallot landraces were used. Japanese LDO varieties are mainly cultivated in northern Japan (44° N, 142° E) in the Hokkaido area, whereas Japanese SDO varieties are cultivated in southern Japan (34° N, 134° E) in the Awaji and Saga areas (Figure 1A,B). LDO varieties are cultivated in March and harvested in early September, whereas SDO varieties are cultivated in early September and harvested by the end of April (Figure 1A,B). On the other hand, all shallot landraces were cultivated on Java Island (6° S, 106° E), Indonesia [11], which is characterized by its Regosol soil type. Both ‘Probolinggo’ and ‘Thailand’ were cultivated in East Java, with a severe dry season, and other cultivars were cultivated in Bantul, Yogyakarta, in the south-central part of Java Island [11]. The cultivation time starts in May, and plants are harvested in July (55–60 days after transplanting) after they have reached full maturity, which is indicated by yellowing of the leaves. Manual irrigation and inorganic fertilizers were applied during the cultivation period every week. All eight Indonesia shallot landraces were collected directly from farmers. The skin was peeled before drying, and bulb tissues of shallots and bulb onions [3 replicates × 3 genotypes (shallot, LDO, and SDO)] were freeze-dried using TAITEC VD-250R freeze dryer coupled with a vacuum pump with ultimate pressure below 50Pa and temperature control at −45 °C. The freeze-dried samples were ground using a small blender, and the dry powders were stored at −21 °C before analysis. 

### 2.2. Metabolome Analysis

The sample preparation process was performed automatically by a liquid handling system (Microlab Star Plus, Hamilton, NV, USA) for dispensing, plate transfer, solvent drying, dissolving, and filtration as described by Sawada et al. [16] and Abdelrahman [4]. Briefly, 4 mg dry weight of bulb tissue powders was accurately weighted and transferred into a 1.5 mL tube with a 3 mm Zirconia bead. The lyophilized powdered bulb onion samples were extracted using 1 mL extraction solvent (80% methanol and 0.1% formic acid coupled with 8.4 nmol L^−1^ lidocaine and 210 nmol L^−1^ 10-camphorsulfonic acid as internal standards) using a multi-bead shocker (Shake Master NEO, Bio Medical Science, Tokyo, Japan) at 1000 rpm for 2 min to obtain final concentration of 4.0 mg mL^−1^. After centrifugation, the extracts were diluted to 400 µg mL^−1^ using an extraction solvent. 25 µL of the extract was transferred to a 96-well plate, dried, redissolved in 250 µL of ultra-pure water (LC-MS grade), and filtered using 0.45 µm pore size filter plates 384 (Multiscreen HTS 384-Well HV, Merk, Rahway, NJ, USA). One microliter of the solution extract at a final concentration of 40 ng μL^−1^ was subjected to widely targeted metabolomics using LC-QqQ-MS (UPLC-Xevo TQ-S, Waters, Milford, MA, USA). The setting parameters for LC–QqQ-MS analysis are summarized in Supplementary Table S7. For ACSO compounds, the freeze-dried powder samples were extracted using 500 µL Mil-li-Q water. After centrifugation, the extracts were diluted 20 times with Milli-Q water and filtrated using 0.45 µm pore size microfilter. The sample extracts were analyzed by 3200 Q TRAP LC-MS/MS using Agilent 1200 Series gradient HPLC system (Agilent Technologies, Santa Clara, CA, USA) equipped with AQUASIL SS-1251-120 column (4.6 mm × 250 mm, Sensyu scientific co., Ltd., Tokyo, Japan) and 3200 Q TRAP mass spectrometer (AB SCIEX LLC, Framingham, MA, USA) with ESI ion source. 0.1% trifluoroacetic acid (TFA) aqueous was used as solvent system under isocratic elution mode and mass range was adjusted at *m*/*z* 5-1700-quad mode. ACSO standards for each compound were used for quantitative and qualitative analysis.

### 2.3. Data Analysis

In total, 499 metabolite intensities were obtained via LC-QqQ-MS analysis. After the missing values were set to 10, the signal intensities of each experimental group were averaged in individual metabolites. The metabolites with signal-to-noise ratio (S/N, defined as the ratio of the average signal intensity to that of the extraction solvent control) <5 in all experimental groups were removed. In addition, the metabolites with a relative standard deviation (RSD) >0.30 in all experimental groups were removed, leaving 172 metabolites for further analysis. The intensities of the 172 metabolites were normalized by dividing the metabolite intensities by the internal standards, and the resulting data matrix was used for comparative analysis (Supplementary Table S1). Initially, all 172 metabolites were subjected to PLS-DA, VIP, and random forest analysis using MetaboAnalyst 5.0 to evaluate the general trend in metabolomic data for the different genotypes. The false-discovery rate (FDR) approach was implemented as a cutoff threshold to identify increased and decreased metabolites [log_2_ (fold changes) ≥1 and ≤−1 with *q*-values ≤ 0.05, respectively] in the “shallot vs. SDO,” “shallot vs. LDO,” and “LDO vs. SDO” comparisons. All the significantly (*p* < 0.05) increased and decreased metabolites in the different comparisons were used to generate volcano plots, Venn diagrams, Pearson’s correlations, and heatmap hierarchal clustering using R v. 4.2.1 (https://www.r-project.org (accessed on 5 July 2022)). The metabolite–metabolite interaction networks were extracted from the search tool for interactions of chemicals (STITCH) [17].

## 3. Results

### 3.1. Characteristic Phenotyping of Shallots and Bulb Onions

A high variability is present among shallot landraces and Japanese LDO and SDO varieties in phenotypes, and the differentiation of the two groups is based on the variation in bulb size, bulb shape, and number of clusters (Figure 1A–C). Shallots have more diverse bulb shapes—ovate, broad oval, and spindle, depending on the variety—while bulb onions mostly have a globe shape (Figure 1A,B). Bulb onions produce only a single bulb per plant, while shallots produce aggregate lateral bulbs. However, the number of lateral bulbs in shallots varies between two and many. Japanese LDO varieties are mainly cultivated in northern Japan, in the Hokkaido area, whereas Japanese SDO varieties are cultivated in southern Japan, in the Awaji and Saga areas (Figure 1A,B). On the other hand, all shallot landraces were cultivated in the lowland of Java Island, Indonesia [11]. Both ‘Probolinggo’ and ‘Thailand’ were cultivated in East Java, which has a severe dry season, and other cultivars were collected from Bantul, Yogyakarta [11]. With respect to bulb size, the diameters of Japanese LDO and SDO bulbs were significantly larger than those of Indonesian shallots, as shown in Figure 1C. Additionally, SDO varieties showed an insignificant increase in bulb diameter compared with LDO varieties (Figure 1C). Additionally, all Japanese LDO and SDO varieties used in this study exhibited yellow color, whereas all the investigated shallot landraces showed pink color (Figure 1A–C). It worth noting that, most of the commercial Japanese LDO and SDO varieties are yellow, which is mainly due to customer preference. 

### 3.2. Comprehensive Metabolite Profiling of Shallots and Bulb Onions

**Figure 2:**
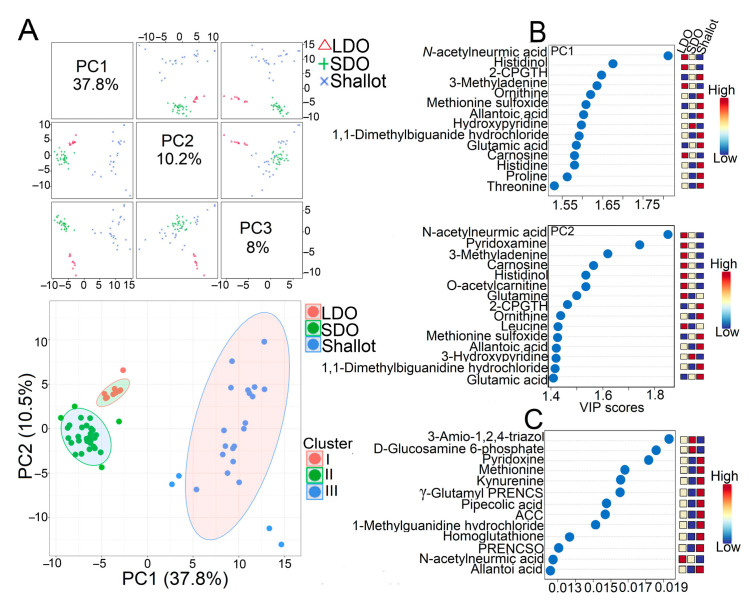
PLS-DA: Partial least-discrimination analysis, VIP: Variable Importance in Projection and Random Forest modeling of the interrelated changes in the metabolite pro-files of Indonesian shallot landraces and Japanese long-day and short-day onions (LDOs and SDOs, respectively). (**A**) PLS-DA Score plot of all 172 variable metabolites identified in the investigated genotypes grown under normal conditions. (**B**) The contribution of each metabolite to PC1 or PC2 axes was colored based on the contribution color scale according to VIP score. (**C**) Random forest variable importance plot. Mean decrease accuracy is the measure of the performance of the model and higher value indicates the importance of that metabolite in predicting group. 2CPGTH, S-2 carboxypropylgluthathione; ACC, 1-aminocyclopropane 1-carboxylic acid; PRENCSO, propenyl-L-cysteine sulfoxide; principal component, PC.

In this study, we used a targeted metabolome profile to investigate the metabolite characteristics of eight Indonesian shallot landraces, three Japanese LDO varieties, and seven Japanese SDO varieties. The Japanese LDO and SDO varieties were cultivated under similar conditions except for the photoperiod requirements. Bulb tissues from each genotype were collected for comprehensive primary and secondary metabolite analyses using LC-QqQ-MS. We obtained 499 metabolite intensities via selected reaction monitoring (SRM) of LC-QqQ-MS. The metabolite data matrix was further filtered by removing the metabolites with S/N < 5 and RSD > 0.30, leaving 172 metabolites for further analysis (Supplementary Table S1). Next, all 172 metabolites were evaluated using supervised multivariate partial least squares discriminant analysis (PLS-DA), variable importance in projection (VIP), and random forest modeling to gain further insight into the contribution of various metabolites in shallots, LDOs, and SDOs (Figure 2A,B). The PLS-DA divides the examined genotypes into three clusters: all Japanese LDO varieties were grouped in cluster I, Japanese SDO varieties in cluster II, and Indonesian shallots in cluster III (Figure 2A). All 172 metabolites were loaded into two major principal components (PC1 and PC2), explaining 48.3% of the total variance (Figure 2A). PC1 separated Indonesian shallot landraces from Japanese SDO and LDO varieties and explained a large proportion (37.8%) of the variance (Figure 2A). On the other hand, PC2 separated Japanese LDO and SDO varieties and explained the lower proportion (10.5%) of variance (Figure 2A). To identify the metabolite variables associated with genotype separation, we carried out VIP and random forest modeling (Figure 2B). The VIP score suggested that several amino acids (e.g., ornithine, histidine, proline, and threonine), organic acids (e.g., glutamic acid and allantoic acid), and sulfur-containing compounds [e.g., S-2-carboxypropylgluthathione (2CPGTH) and methionine sulfoxide] were highly associated with Indonesian shallot landraces (Figure 2B). Meanwhile, N-acetylneuramic acid/sialic acid, pyridoxamine/vitamin B6, carnosine, histidinol, O-acetyl-carnitine, glutamine, and leucine were more associated with LDO varieties, whereas only hydroxypyridine was associated with SDO varieties (Figure 2B). Random forest modeling suggested that several sulfur-containing compounds [e.g., gamma-Glu-PREN and propenyl-L-cysteine sulfoxide (PRENCSO)] and amino acids (e.g., methionine, kynurenine, tryptophan, homoglutathione, and allantoin) were associated with Indonesian shallots (Figure 2B). On the other hand, N-acetylneuramic acid was mainly associated with LDO varieties, whereas amino-1,2,4 triazole and glucoseamine-6-phosphate were more closely associated with SDO varieties (Figure 2B). The results of PLS-DA, VIP, and random forest modeling collectively indicated that shallot landraces and Japanese LDO varieties are richer in metabolite constituents in comparison with SDO varieties. In addition, amino acid and sulfur-containing compounds are the key characteristic metabolites in shallots and LDO varieties (Figure 2B).

**Figure 3:**
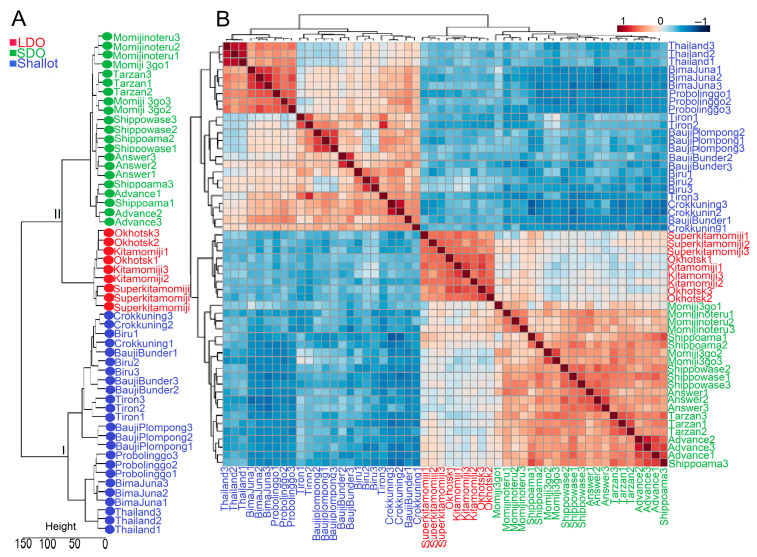
Dendrogram clustering and Pearson correlation heatmap of the investigated Indonesian shallot landraces and Japanese long-day and short-day onions (LDOs and SDOs, respectively) genotypes grown under normal growth conditions. (**A**) Dendrogram clustering of the investigated genotypes using 172 metabolite variables. The height of the bar indicates the distance between the clusters. (**B**) Heatmap of the Pearson correlation of the investigated genotypes based on metabolite variables. Color scale indicates Pearson correlation coefficients.

To validate the results obtained by PLS-DA, we conducted dendrogram clustering analysis of all investigated genotypes using the 172-metabolite data matrix (Figure 3A). As in the PLS-DA, the investigated genotypes were separated into two major clusters (Figure 3A). Cluster I contained all Indonesian shallot landraces, whereas cluster II was divided into two sub-clusters, II.1 and II.2, containing Japanese LDO and SDO varieties, respectively (Figure 3A). Additionally, Pearson correlation analysis revealed a positive relationship (r = 0.04 to 0.84) among all shallot landraces except ‘Tiron’, ‘BaujiPlompong’ and ‘Thailand’ which exhibited weak negative relationships (r = −0.05 to −0.12) among each other (Figure 3B). On the other hand, LDO varieties showed positive relationships (r = 0. 38 to 0.70) with each other (Figure 3B). Likewise, all SDO varieties exhibited positive relationships (r = 0.08 to 0.79) among each other (Figure 3B). On the other hand, Indonesian shallots displayed negative relationships (r = −0.003 to −0.63) with Japanese LDO and SDO varieties (Figure 3B). Genotype correlation analyses provide additional evidence regarding the variations in metabolite profiles of the investigated genotypes, and shallots have distinct metabolite profiles compared with LDO and SDO varieties.

### 3.3. Genotype Correlation and Venn Diagram Analysis of Shallots and Bulb Onions

**Figure 4:**
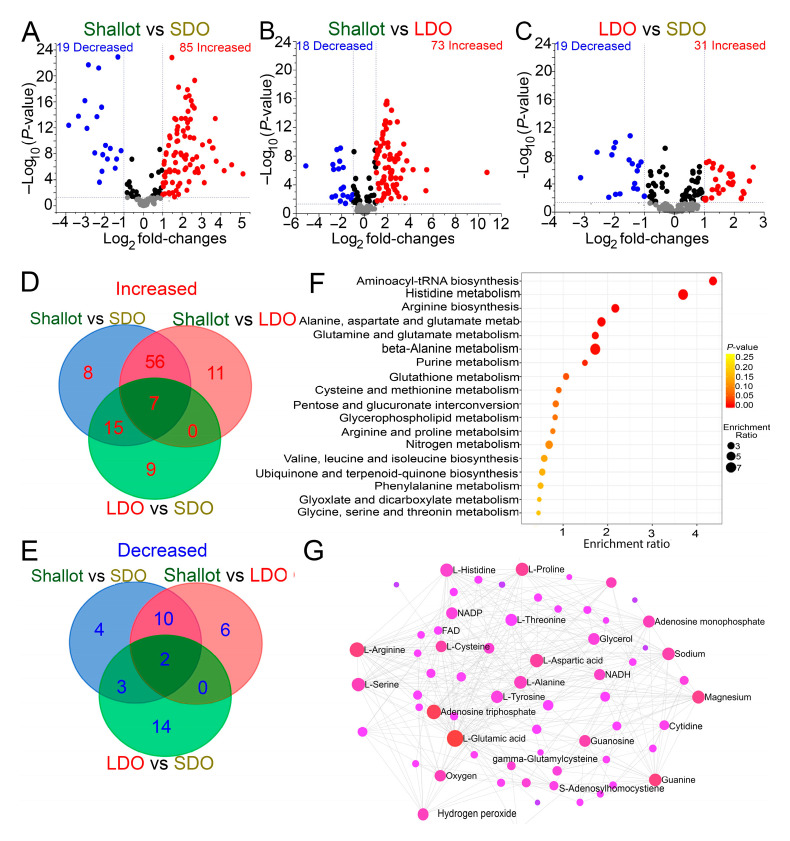
Volcano plots, Venn diagrams, pathway enrichment and metabolite-metabolite interaction networks of differentially produced metabolites (DPMs) in Indonesian shallot landraces and Japanese long-day and short-day onions (LDOs and SDOs, respectively), under normal growth conditions. (**A**–**C**) Volcano plots of significantly increased [log_2_ (fold-changes) ≥ 1; *q*-values ≤ 0.05] and decreased [log_2_ (fold-changes) ≤ −1; *q*-values ≤ 0.05] metabolites in the investigated comparisons. Blue-dashed lines represent the q-value and fold-change threshold. Red and blue points highlight the increased and decreased metabolites, respectively, in the investigated comparisons. (**D**,**E**) Venn diagrams of overlapping increased (**D**) and decreased (**E**) metabolites in ‘shallot vs. SDO’, ‘shallot vs. LDO’ and ‘LDO vs. SDO’ comparisons. (**F**) Pathway enrichment analyses of metabolites differentially produced in ‘shallot vs. SDO’, ‘shallot vs. LDO’ and ‘LDO vs. SDO’ comparisons. Colors of the circles indicate high and low −log_10_ (*p*-values), respectively. The circle size indicates a high (larger circle) or low (smaller circle) enrichment ratio. (**G**) Metabolite-metabolite interaction networks. The size and color indicate high metabolite-metabolite interactions.

The FDR approach was implemented to identify the differentially produced metabolites (DPMs) in the Indonesian shallots and Japanese LDO and SDO varieties under normal growth conditions. Among the 172 identified metabolites, 85, 73, and 31 metabolites increased [log_2_ (fold changes) ≥ 1; *q*-values ≤ 0.05], whereas 19, 18, and 19 metabolites decreased [log_2_ (fold changes) ≤ −1; *q*-values ≤ 0.05] in the “shallot vs. SDO,” “shallot vs. LDO,” and “LDO vs. SDO” comparisons, respectively (Figure 4A–C; Supplementary Tables S1–S3). These results collectively indicate that in terms of the number of altered metabolites, a greater metabolic change occurred under normal growth conditions, when shallots were compared with Japanese LDO or SDO varieties, while a smaller metabolic change occurred in the “LDO vs. SDO” comparison. These results confirmed that shallots, followed by LDOs, are richer in metabolite constituents compared with SDO varieties. We identified the overlapping and specific DPMs in shallots and Japanese LDO and SDO varieties grown under normal growth conditions based on the construction of a Venn diagram (Figure 4D,E; Supplementary Table S6). In total, 7 and 2 overlapping increased and decreased metabolites, respectively, were detected in “shallot vs. SDO,” “shallot vs. LDO,” and “LDO vs. SDO” comparisons (Figure 4D,E; Supplementary Table S6), whereas 56 and 10 overlapping increased and decreased metabolites, respectively, were detected in “shallot vs. SDO” and “shallot vs. LDO” comparisons (Figure 4D,E; Supplementary Table S6). The identified 66 (56 increased and 10 decreased) overlapping DPMs in “shallot vs. LDO” and “shallot vs. SDO” comparisons indicated that shallot landraces exhibited a constitutive accumulation of or decrease in these metabolites independent of LDO and SDO varieties under normal growth conditions, and these 66 metabolites represent the core characteristic metabolites in shallot landraces. Notably, 15 and 3 increased and decreased metabolites, respectively, were observed in “shallot vs. SDO” and “LDO vs. SDO” varieties (Figure 4D,E; Supplementary Table S6). In contrast, 9 and 14 increased and decreased metabolites, respectively, were detected in the “LDO vs. SDO” comparison (Figure 4D,E; Supplementary Table S6). The identified 23 (9 increased and 14 decreased) metabolites in the “LDO vs. SDO” comparison indicated that LDO varieties exhibited a constitutive increase or decrease in these metabolites independent of shallots and SDOs under normal growth conditions, and these 23 metabolites represent the core characteristic metabolites in LDO varieties. Thus, it is plausible that the 89 (66 and 23) identified DPMs may be the key metabolites playing an integral role in the genotype differences exhibited by shallot and LDO genotypes, respectively (Supplementary Table S6).

### 3.4. Metabolic Pathways Associated with Shallots and Bulb Onions

A metabolite pathway–enrichment analysis was conducted based on the Arabidopsis Kyoto Encyclopedia of Genes and Genomes (KEGG) database to identify metabolic pathways associated with the 89 overlapping DPMs (Figure 4F). These results indicated that most of the DPMs were enriched in the pathways associated with aminoacyl-tRNA biosynthesis, histidine metabolism, arginine biosynthesis, glutamine and glutamate biosynthesis, beta-alanine metabolism, purine metabolism, and glutathione metabolism (Figure 4F). The pathway-enrichment analysis of the 89 overlapping DPMs in shallot landraces and LDO varieties indicated that most of the metabolites are highly involved in the amino acid and organic acid–related pathways (Figure 4F). Next, a metabolite–metabolite interaction network analysis was carried out to highlight the potential functional relationships among 89 overlapping DPMs in an intuitive way (Figure 4G). The chemical–chemical associations for the metabolite network were extracted from STITCH [17], where only highly confident chemical–chemical interactions are selected (Figure 4G). Our results indicated that organic acids, such as glutamic and aspartic acids, showed high chemical–chemical interactions, implying that these molecules may play an important role(s) in shallot landraces and LDO varieties (Figure 4G). Specifically, glutamic acid plays an integral role in organosulfur compound biosynthesis [18]. In addition, several amino acids, such as arginine, proline, histidine, serine, and alanine, also showed high chemical–chemical associations in the metabolite network (Figure 4G). Metabolite–metabolite interaction network results were consistent with the pathway-enrichment analysis, where amino acids and organic acids were enriched in the shallot and LDO varieties (Figure 4F,G).

### 3.5. Comparative CSO Biosynthesis Pathway in Shallots and Bulb Onions

**Figure 5:**
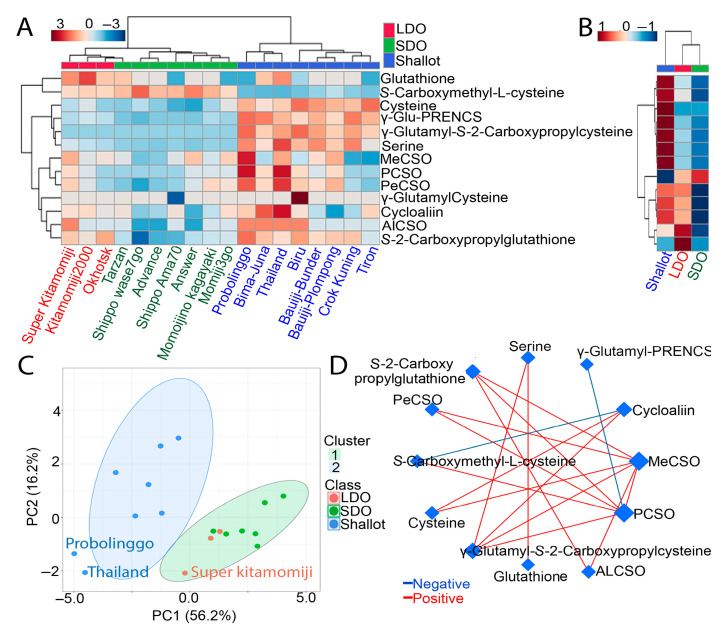
Heatmaps, principal component analysis (PCA) and correlation network of the investigated Indonesian shallot landraces and Japanese long-day and short-day onions (LDOs and SDOs, respectively), based on organosulfur compounds. (**A**,**B**) Heatmaps of the normalized organosulfur compound contents in the investigated genotypes. Color bar indicates high (red) and low (blue) contents of organosulfur compounds. (**C**) PCA-score plot of the investigated genotypes based on organosulfur compounds. (**D**) Metabolite-metabolite correlation using Pearson correlation coefficient.

Since ACSOs are the main characteristics and active metabolites in shallot and onion cultivars, we measured the absolute contents of several down- and upstream metabolites associated with the CSO pathway (Figure 5A,B). In general, most of the down- and upstream metabolites associated with the ACSO pathway displayed high accumulation in shallot landraces compared with Japanese LDO and SDO varieties, except for glutathione and S-carboxymethyl-L-cysteine (Figure 5A,B). In addition, Japanese LDO varieties exhibited a higher accumulation of ACSO-related metabolites compared with SDO varieties (Figure 5A,B). Specifically, ‘Super Kitamomiji’, followed by ‘Kitamomiji’, showed higher ACSO-related metabolite content compared with other LDO and SDO varieties (Figure 5A,B). To confirm the variation between shallot landraces as well as Japanese LDO and SDO varieties with respect to ACSO-related metabolites, we carried out a principal component analysis (PCA, Figure 5C). All 13 ACSO-related metabolites were loaded into two major principal components, PC1 and PC2, explaining 72.4% of the total variance (Figure 5C). Indonesian shallot landraces, as well as Japanese SDO and LDO varieties, were mainly separated by PC1, which explained a large proportion of the variance (56.2%), while the lower proportion of variance (16.2%) was explained by PC2 (Figure 5C). The PCA divides the examined genotypes into two clusters: all Japanese LDO and SDO varieties were grouped in cluster I, whereas all Indonesian shallots were grouped in cluster II (Figure 5C). Heatmap clustering and PCA collectively indicated that shallot landraces, followed by Japanese LDO varieties, have more ACSO-related metabolites, whereas Japanese SDO varieties have the lowest amount (Figure 5A–C). In support of the above results, the correlation network analysis based on the partial least squares coefficient showed high positive relationships (r = 0.99 to 1.0) among all ACSO-related metabolites except for cycloalliin, S-carboxymethyl-L-cysteine, propyl-l-cysteine sulfoxide (PCSO), and γ-glutamyl PRENCS (Figure 5D). For example, a negative relationship (r = −1.0) was observed between cycloalliin and S-carboxymethyl-L-cysteine, and another negative relationship (r = −1.0) was detected between PCSO and γ-glutamyl PRENCS (Figure 5D).

**Figure 6:**
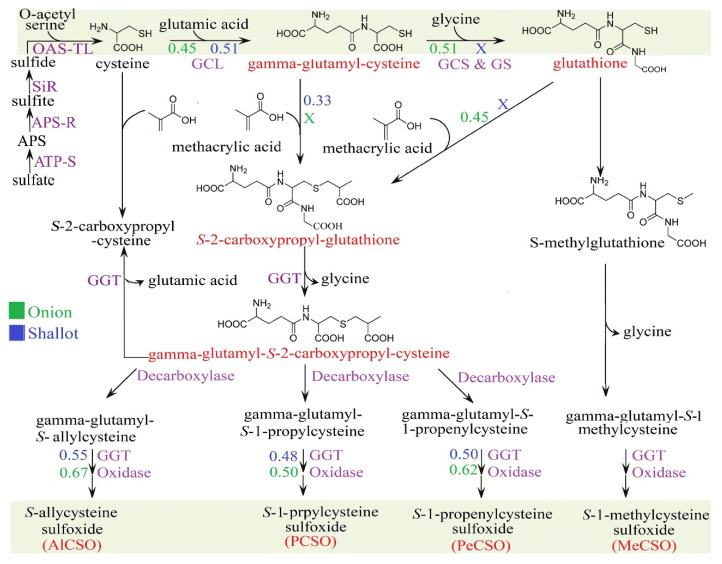
Putative pathways for the biosynthesis of S-alk(en)yl-L-cysteine sulfoxide compounds in shallots and bulb onions. The sulfur assimilation pathway in onion begins with the activation of sulfate (SO_4_^2−^) to adenosine 5′-phosphosulfate (APS), a reaction catalysed by ATP-sulfurylase (ATP-S). APS is reduced to sulfide (S^2−^) in a two-step process catalysed by the enzymes APS-reductase (APS-R) and sulfite reductase (SiR). The generated S^2−^ incorporated into cysteine, a donor of reduced sulfur for all further S-containing metabolites. Biosynthesis of *S*-alk(en)yl-l-cysteine sulfoxides is initiated by *S*-alk(en)ylation of glutathione, which is followed by the removal of glycyl and γ-glutamyl groups and S-oxygenation. Values represent Pearson correlation coefficients of the investigated genotypes. GCL, gamma-glutamylcysteine synthetase; GS, glutathione synthetase; GGT, gamma-glutamyl transpeptidase; OAS-TL, *O*-acetylserine (thiol)-lyase.

Next, we depicted the biosynthesis pathway of ACSO based on the literature [18,19] and the KEGG database (Figure 6) to identify the variation in the ACSO biosynthesis pathway between shallots and bulb onions. Based on Pearson correlation analysis of the measured metabolites in shallot and bulb onion varieties, we found that there were no significant differences in the upper-stream pathway between cysteine and γ-glutamyl-cysteine in bulb onions and shallot landraces; the Pearson correlation coefficients were 0.45 and 0.51, respectively (Figure 6). Interestingly, bulb onion varieties showed good correlation (r = 0.51) between γ-glutamyl-cysteine and glutathione, whereas no correlation was observed in shallot landraces (Figure 6). In this study, a positive correlation (r = 0.46) was observed in bulb onion varieties between glutathione and S-2-carboxypropyl glutathione, whereas no correlation was observed in shallot landraces (Figure 6). In contrast, we observed a weak positive relationship (r = 0.33) between γ-glutamyl-cysteine and S-2-carboxypropyl glutathione in shallot landraces, whereas no correlation was observed in bulb onion varieties (Figure 6). The above results highlight the possibility of a short route in shallots for ACSO biosynthesis. Specifically, shallot landraces showed positive correlations between γ-glutamyl-cysteine and S-2-carboxypropyl glutathione (Figure 6), which escape the regular route that includes the conversion of γ-glutamyl-cysteine to glutathione, followed by the conversion of glutathione to S-2-carboxypropyl glutathione (Figure 6). This short route might be activated in shallot landraces to save energy under stress conditions, especially in those grown mainly in tropical areas with warm conditions and a high possibility of pathogen infection [5]. However, further investigation using radio labeling techniques must be conducted to validate our hypothesis. 

## 4. Discussion

Onions and shallots are both bulb vegetables in the same *Allium* family that likely became domesticated in Southwest or Central Asia [20,21]. Onions and shallots are both used as ingredients to flavor food dishes and can be eaten on their own; thus, shallots are yin to the onion’s yang. However, shallots and onions are different in phenotype and metabolite constituents [7]. For example, onions form one large, single bulb, while shallots grow in clusters of small bulbs (Figure 1A,B). Shallots accumulate higher amounts of amino acid, carbohydrate, phospholipid, flavonoid, and ACSO [especially methiin (MeCSO) and isoalliinl/PRENCSO (PeCSO)] content than bulb onions, which explain the high pungency of shallot landraces [5,9,11]. Thus, further investigation of the metabolic characteristics of shallots and bulb onions is needed to select superior varieties for the development of a novel F_1_ hybrid between shallots and bulb onions with better taste and higher adaption to environmental stresses. The top three bulb onion-producing areas in Japan are Hokkaido (the northernmost island), Hyogo (a district of west central Japan), and Saga (a district in southwestern Japan). In 2018, bulb onion production in Hokkaido accounted for 62.1% of national output, followed by Saga (10.2%) and Hyogo (8.3%) [22]. Over the past three decades, open-pollinated USA cultivars have been replaced with Japanese F_1_ hybrid cultivars that offer higher yield, better disease resistance, and high bulb quality [23]. Briefly, foreign bulb onion varieties introduced in the early Meiji period, and trials for full-scale cultivation beginning in Hokkaido and Osaka. The variety was introduced to Sapporo in 1871, and through repeated selection and cultivation trials, it was transformed into the native variety ‘Sapporo Ki’. In 1920, ‘Sapporo Ki’ was introduced to Aiho-Futajima district, Yamaguchi City, where it was cultivated under autumn sowing conditions. The short-day storable variety ‘Yamaguchi Kodaka (Yamaguchi Maru)’ was bred. On the other hand, the USA pungent onion variety ‘Yellow Danvers’ suitable for storage was selected for fall sowing in the Sennan area of Osaka from 1879, and ‘Senshu Yellow’ was born. At the same time, the French autumn-sown hot onion variety ‘Blanc à Chief de Paris’ was introduced from France, and its cultivation and selection began in the Chita Peninsula of Aichi Prefecture in the early Taisho period, leading to the birth of the extremely early maturing white variety ‘Aichi Shiro’. In general, ‘Sapporo Ki’ and ‘Yamaguchi Kodaka’, two of the above four varieties, became the foundation of Japanese yellow onion F_1_ breeding materials together with a male sterile line ‘W202A’ from USA [24]. Currently, F_1_ hybrid cultivars developed by private seed companies occupy large onion-growing areas in Japan, though the accurate numerical data for acreage and production under hybrid bulb onions are not available [24]. Specifically, two groups of Japanese LDO and SDO varieties differentiated by their photoperiod requirements for bulb formation were examined in this study. The Japanese SDO needs 10–11 h of daylight, while the LDO needs 14–16 h of daylight for bulb formation [15]. Although previous studies indicated that different photoperiods did not affect bulb yield in LDO and SDO varieties, the dry matter contents in LDO varieties were slightly higher than those in SDO varieties [11,25]. However, there is no information about the differences in metabolic profiles between LDO and SDO varieties. In this study, we investigated the metabolic profiles of Japanese LDO and SDO varieties and Indonesian shallot landraces to identify genotype-specific metabolites and to identify superior varieties for the *Allium* breeding program.

### 4.1. Comparative Metabolome Analyses of Shallot and Bulb Onions

Analysis of the metabolome data for shallots and bulb onions under normal growth conditions using PLS-DA, VIP, random forest modeling, and Pearson correlation revealed a distinct metabolic profile of shallot landraces compared with LDO and SDO varieties (Figure 2A,B and Figure 3A,B; Supplementary Table S1). In addition, LDO varieties were separated from SDO varieties, implying that different photoperiods and genetic background affected the metabolite profiles of the investigated bulb onion genotypes (Figure 2A and Figure 3A; Supplementary Table S1). Our results indicated that amino acids, organic acids, sugars, and organosulfur compounds are key metabolites responsible for genotype separation (Figure 2A and Figure 3A). Specifically, the analyses of 56 increased and 10 decreased metabolites identified in “shallot vs. SDO” and “shallot vs. LDO” comparisons indicate that the accumulations of metabolites related to the organic acid, sugar, amino acid, flavonoid, nucleoside, and sulfur metabolism are primarily detected in the shallot landraces (Figure 4D,E; Supplementary Table S5). With respect to LDO varieties, the Venn diagram analysis of 9 increased and 14 decreased metabolites identified in “LDO vs. SDO” indicates that the accumulation of metabolites related to organic acid, amino acid, and flavonoid metabolism and the decrease in metabolites related to nucleoside and sugar metabolism are primarily detected in LDO varieties (Figure 4D,E; Supplementary Table S5). These data indicate that the shallots placed the metabolism of these metabolites at a stage ready to react to unfavorable conditions in the tropical environment [9,11]. However, further investigation of these Japanese LDO and SDO varieties and shallot landraces under conducive environment stress is needed to validate this metabolic adaption mechanism. The influence of environmental factors on bulb formation and true seed production varies greatly among shallots, and thus shallots reprogram their metabolism to overcome the adverse effects of environmental stresses to produce seeds and bulbs with high yield and quality [26,27,28]. Shallot is also preferred for its shorter growth cycle, better tolerance to disease and drought stresses and longer storage life than the common onion [29]. Taking the influence of genetic characteristics into consideration, the selection of appropriate varieties with high adaptability to harsh environmental conditions can promote the yield and quality of shallot seeds and bulbs [30]. In addition, long-day cultivation induces these metabolic pathways in LDO varieties to synchronize their developmental process and alleviates the impacts of environmental stresses. Photoperiod changes can affect the metabolite profiles of plants [31], which enables them to synchronize their developmental processes with a specific time of year and alleviates the impact of environmental stresses occurring at the same time every year [32,33]. For example, the photoperiod has been shown to influence the plants’ responses to drought stress [34], salt stress [35], and pathogens [36]. Collectively, these findings indicate that changes in the metabolism of certain amino acid, organic acid, flavonoid, and sulfur-related pathways are characteristic features of shallot and LDO varieties, which might be helpful in plant adaptation to a tropical environment and long-day cultivation, respectively.

### 4.2. Comparative Analysis of the Sulfur Assimilation Pathway in Shallots and Bulb Onions

Organosulfur compounds are major sinks for assimilated sulfate (SO_4_^2−^) in onion and accumulation varies widely depending on plant genotype and sulfur supply [37]. Although the molecular basis for the genetic variation is not fully understood, physiological studies have revealed significant differences in the uptake, assimilation and partitioning of sulfate and organic sulfur compounds between low- and high-pungent onion varieties [38]. For example, high-pungent onion genotype ‘W202A’ and low-pungent genotype ‘Texas Grano 438’ grown hydroponically under deficient (-S) and sufficient (+S) conditions, showed higher levels of total S and ACSO precursors in pungent ‘W202A’ under +S condition [38]. In addition, the ‘W202A’-pungent genotype displays significantly higher cysteine content in the leaves relative to low-pungent ‘Texas Grano 438’ genotype [38]. Significant ‘genotype × S’ treatment effects were observed in root *high affinity-sulfur transporter* (*HAST*) and *ferredoxin-sulfite reductase* (*SiR*) genes. Specifically, *ATP-sulfurylase* (*ATP-S*) transcript levels were significantly higher in pungent ‘W202A’ genotype and +S condition [38]. ATP-S catalyzes SO_4_^2−^ activation and yields activated high-energy compound adenosine-5′-phosphosulfate that is reduced to sulfide (S^2−^) and incorporated into cysteine. In turn, cysteine acts as a precursor or donor of reduced-S for a range of S-compound biosynthesis [39]. Today, the biosynthetic pathway of ACSO has become much clearer despite some of its details remain under debate [40]. Early studies by Lancaster and Shaw [41] and Randle et al. [42] have proposed a putative pathway via glutathione *S*-conjugates based on pulse radiolabeling studies of *Allium* tissues. In the proposed pathway, glutathione is *S*-alk(en)ylated at the cysteine residue, followed by the removal of a glycyl group to form a biosynthetic intermediate, γ-glutamyl-*S*-alk(en)yl-L-cysteine. This γ-glutamylated sulfide compound is further deglutamylated and *S*-oxygenated to yield ACSO [18,19]. Although the results of pulse radiolabeling suggest that *S*-oxygenation may likely occur before deglutamylation in onion [41], the order of *S*-oxygenation and deglutamylation in other *Allium* plants remains unclear [19]. It can be speculated that the intermediate γ-glutamyl-*S*-allyl-L-cysteine is mainly deglutamylated prior to being *S*-oxygenated in Alliin biosynthesis in garlic [18,19]. This hypothesis is also supported by recent study on flavin-dependent *S*-oxygenase (FMO), which preferably utilizes *S*-allyl-L-cysteine, rather than γ-glutamyl-*S*-allyl-L-cysteine, as the substrate [18,19]. One plausible pathway for the biosynthesis of ACSO involves biosynthesis of *S*-2-carboxypropyl glutathione from glutathione and methacrylic acid [18,40]. Whereas another rout involves the biosynthesis of *S*-2-carboxypropyl glutathione from γ-glutamyl cysteine without passing through glutathione (Figure 6). An alternative biosynthetic pathway omits glutathione in favor of direct alk(en)ylation of cysteine or thioalk(en)ylation of O-acetyl serine followed by oxidation to a sulphoxide [18,19]. In both pathways, few of the proposed biosynthetic enzymes from Alliums have been studied in detail, however, tissue specific enzymes and the source of the alk(en)yl groups in both pathways remains to be resolved [18,19]. For example, the elimination of glycine and glutamic acid from *S*-2-carboxypropyl glutathione by γ-glutamyl transpeptidase (GGT) produces *S*-2-carboxypropyl cysteine (Figure 6), which is converted to *S*-allyl cysteine by decarboxylation and oxidation-related genes [18,41]. *S*-Allyl cysteine is also biosynthesized via the elimination of glutamic acid from γ-glutamyl *S*-allyl cysteine by GGT [18]. Finally, the sulfur oxidation of *S*-allyl cysteine by flavin-containing monooxygenase produces ACSO [18,19]. 

In this study, we focused on organosulfur compounds as main active ingredients responsible for many nutritional properties in shallots and bulb onions [43,44,45]. Organosulfur compounds, known as flavor precursors, contributed to the pungent taste of shallots and bulb onions [15,46,47]. Specifically, MeCSO and PeCSO were found to be the main organosulfur compounds correlated to the pungency of shallots and bulb onions [48]; however, AlCSO was found at low concentrations or even was undetected [49,50]. In this study, many of the organosulfur compounds, such as MeCSO, PCSO, PeCSO, AlCSO, γ-glutamyl PRENCS, γ-glutamyl-*S*-2-carboxypropylcysteine, and S-2-carboxypropylglutathione, were highly accumulated in shallot landraces, especially ‘Probolinggo’ and ‘Thailand’ (Figure 5A,B). Additionally, LDO varieties, especially ‘Super Kitamomiji’, exhibited higher MeCSO, PCSO, PeCSO, AlCSO, and *S*-carboxymethyl-L-cysteine contents than SDO varieties (Figure 5A,B). Interestingly, *S*-carboxymethyl-L-cystine seems to be uniquely accumulated in LDO and SDO varieties but is low in shallots (Figure 5A,B). Our results were consistent with those of Ariyanti et al. [11], who reported high levels of MeCSO and PeCSO in shallot landraces with a strong pungent taste. Likewise, LDO varieties cultivated during the summer season, from March to September, in northern Japan had a higher PeCSO content than SDO varieties mainly grown in the winter season, from September to April [11]. Our results and previous reports indicated that the difference in photoperiod and genetic background may partially affect the sulfur assimilation and subsequently the organosulfur compound content. For example, early studies suggested that the organosulfur compound amounts are affected by the variety, maturity, soil fertility, and other growing conditions, and the increase in temperature may increase the pungency in shallots and bulb onions [15,51]. In this study, the two shallot landraces, ‘Probolinggo’ and ‘Thailand’, exhibited higher organosulfur compounds than other shallot landraces, which might be partially attributed to some genetic variances and the growth conditions of these two varieties in East Java, Indonesia [11]. Likewise, the LDO ‘Super Kitamomiji’ is mainly cultivated during the summer season in Japan, which induces greater accumulation of organosulfur compounds than SDO varieties. PCA analysis of the investigated genotypes based on organosulfur compounds also revealed a distinct organosulfur profile of shallots relative to LDO and SDO varieties, and ‘Probolinggo’ and ‘Thailand’ were separated from other shallot landraces (Figure 5C). Metabolite–metabolite correlation also showed a highly positive relationship among organosulfur compounds, except the genitive relationship between cycloalliin and *S*-carboxymethyl-L-cysteine (r = −1), and between PCSO and γ-glutamyl PRENCS (r = −0.99) (Figure 5D).

Next, we ran a correlation analysis of the organosulfur compounds in each genotype separately to find the difference in sulfur pathways between bulb onions and shallots (Figure 6). Our results showed positive relationships (r = 0.45 and 0.51) between the cysteine and γ-glutamyl-cysteine we found in bulb onions and shallots, respectively (Figure 6), implying that there are no differences in the upstream sulfur pathway between shallots and bulb onions. On the other hand, positive relationships between γ-glutamyl-cysteine and glutathione (r = 0.51), and between glutathione and *S*-2-carboxypropyl-glutathione (r = 0.45), were found in bulb onions, but no correlations were detected in shallots (Figure 6). In contrast, a weak positive relationship between γ-glutamyl-cysteine and *S*-2-carboxypropyl-glutathione (r = 0.33) was found in shallots, but no correlations were detected in bulb onions (Figure 6). These results suggested that shallots may use a short route for organosulfur biosynthesis through γ-glutamyl-cysteine and *S*-2-carboxypropyl-glutathione, which might reduce energy consumption and enable shallots to survive under stress environments. Our metabolite–metabolite interaction networks revealed a high interaction with ATP, the main energy currency of the cell and signaling molecule for cell communication [52], which indicated that shallots could maintain their energy balance by changing their metabolism.

## 5. Conclusions

Environmental cues play important roles in the regulation of a plant’s physiology and behavior. One such cue, the photoperiod, regulates different morphophysiological processes in plants, directly impacting photosynthetic performance and, consequently, the primary and secondary metabolism. In this study, we used LC-QqQ-MS analysis to find the discriminative metabolites between Japanese LDO and SDO varieties and Indonesian shallots grown under normal conditions. Our results showed different metabolite profiles between shallot landraces and Japanese LDO and SDO varieties. Specifically, shallots displayed a higher accumulation of amino acids, organic acids, and organosulfur compounds relative to LDO and SDO varieties. In addition, LDO varieties showed a higher accumulation of the above-mentioned compounds compared with SDO varieties. Collectively, our results indicated that shallots, followed by LDO varieties, exhibited richer metabolite constituents than SDO varieties. Among the investigated shallots, ‘Probolinggo’ and ‘Thailand’ showed the highest accumulation of organosulfur compounds, whereas ‘Super Kitamomiji’ showed the highest accumulation of organosulfur compounds among LDO varieties. However, the results found in this study only pertain to those varieties evaluated and not to all LDO and SDO varieties grown in other different climatic zones. Future study directed toward the differences in sulfur assimilation pathways between shallots and bulb onions might provide further in-depth information about energy consumption and organosulfur biosynthesis. Finally, a breeding program toward the production of a novel F_1_ hybrid derived from a crossing between ‘Probolinggo’ and ‘Super Kitamomiji’ and between ‘Thailand’ and ‘Super Kitamomiji’ can improve the organosulfur content and enhance the pungency and aroma of onion varieties. In the future, a comparative analysis of organosulfur compounds in different SDO and LDO varieties derived from different regions and climatic zones, may provide useful information for *Allium* breeding to improve taste and aroma. 

## Data Availability

Normalized data from LC-QqQ-MS metabolite profiling are provided in Supplementary Table S1. Additionally, the raw MS data can be downloaded from Drop Met database (http://prime.psc.riken.jp/menta.cgi/prime/drop_index (accessed on 15 March 2022)). The investigated materials are available at Shigyo’s lab, Yamaguchi University, Japan, and can be provided upon request.

## Supplementary Materials

The following supporting information can be downloaded at: https://www.mdpi.com/article/10.3390/metabo12121260/s1, Table S1: List of normalized primary and secondary metabolites successfully identified in Japanese short-day and long-day onion (SDO and LDO, respectively) varieties, and Indonesian shallots grown under normal growth conditions, as determined by liquid chromatography–tandem quadrupole mass spectrometry (LC-QqQ-MS). Normalized data represent three biological replicates (*n* = 3).; Table S2: Fold-changes and false discovery rates (FDR) of differentially produced metabolites (FDR < 0.05) in shallot compared with short-day onion (SDO) varieties. Red color indicates differentially increased [FDR < 0.05, log_2_ fold-changes > 1] and decreased [FDR < 0.05, log_2_ fold-changes < −1] metabolites.; Table S3: Fold-changes and false discovery rates (FDR) of differentially produced metabolites (FDR < 0.05) in shallot compared with long-day onion (LDO) varieties. Red color indicates differentially increased [FDR < 0.05, log_2_ fold-changes > 1] and decreased [FDR < 0.05, log_2_ fold-changes <−1] metabolites.; Table S4: Fold-changes and false discovery rates (FDR) of differentially produced metabolites (FDR < 0.05) in long-day onion (LDO) compared with short-day onion (SDO) varieties. Red color indicates differentially increased [FDR < 0.05, log_2_ fold-changes > 1] and decreased [FDR < 0.05, log_2_ fold-changes < −1] metabolites.; Table S5: List of overlapping differentially produced metabolites [DPMs, *q*-values ≤ 0.05; log_2_ (fold-changes) ≤ −1 and log_2_ (fold-changes) ≥ 1 for decreased and increased metabolites, respectively] in ‘shallot vs. SDO’, ‘shallot vs. LDO’ and/or ‘LDO vs. SDO’ comparisons. Values represent metabolite log_2_ (fold-changes). Red and blue colors indicate overlapping increased and decreased levels of metabolites, respectively, in different comparisons.; Table S6: List of organosulfur-related compounds successfully identified in Japanese short-day and long-day onion (SDO and LDO, respectively) varieties, and Indonesian shallots grown under normal growth conditions, as determined by liquid chromatography–mass spectrometry (LC-MS). Data represent average mean (mg g^−1^ DW) of three replicates (*n* = 3).; Table S7: Setting parameters of LC-QqQ-MS analysis of Indonesian shallots and Japanese long-day and short-day bulb onions.
